# Network-based identification and characterization of teleconnections on different scales

**DOI:** 10.1038/s41598-019-45423-5

**Published:** 2019-06-19

**Authors:** Ankit Agarwal, Levke Caesar, Norbert Marwan, Rathinasamy Maheswaran, Bruno Merz, Jürgen Kurths

**Affiliations:** 1 Potsdam Institute for Climate Impact Research (PIK), Member of the Leibniz Association, Telegrafenberg, Potsdam, Germany; 2 Institute for Environmental Sciences and Geography, University of Potsdam, Potsdam, Germany; 3 GFZ German Research Centre for Geosciences, Section 4.4: Hydrology, Telegrafenberg, Potsdam, Germany; 4 Institute of Physics and Astronomy, University of Potsdam, Potsdam, Germany; 5 MVGR college of Engineering, Vizianagaram, India; 6 Institute of Physics, Humboldt Universität zu Berlin, Berlin, Germany

**Keywords:** Mathematics and computing, Physics

## Abstract

Sea surface temperature (SST) patterns can – as surface climate forcing – affect weather and climate at large distances. One example is El Niño-Southern Oscillation (ENSO) that causes climate anomalies around the globe via teleconnections. Although several studies identified and characterized these teleconnections, our understanding of climate processes remains incomplete, since interactions and feedbacks are typically exhibited at unique or multiple temporal and spatial scales. This study characterizes the interactions between the cells of a global SST data set at different temporal and spatial scales using climate networks. These networks are constructed using wavelet multi-scale correlation that investigate the correlation between the SST time series at a range of scales allowing instantaneously deeper insights into the correlation patterns compared to traditional methods like empirical orthogonal functions or classical correlation analysis. This allows us to identify and visualise regions of – at a certain timescale – similarly evolving SSTs and distinguish them from those with long-range teleconnections to other ocean regions. Our findings re-confirm accepted knowledge about known highly linked SST patterns like ENSO and the Pacific Decadal Oscillation, but also suggest new insights into the characteristics and origins of long-range teleconnections like the connection between ENSO and Indian Ocean Dipole.

## Introduction

The ocean covers more than two-thirds of the Earth’s surface. Given the higher heat capacity of water as compared to air (by approximately a factor of 4) and greater mass in the ocean than in the atmosphere, the ocean can store about 1000 times as much heat as the atmosphere^1^. Although it takes time to transport heat in or out of the ocean, even the ocean mixed-layer has a larger heat capacity than the atmosphere and can, therefore, strongly influence the climatic conditions. As a consequence, the ocean is capable of altering atmospheric conditions on seasonal and longer timescales^2^.

As the sea surface forms a direct boundary between ocean and atmosphere, the sea surface temperature (SST) is of particular importance for setting the atmospheric conditions. In the opposite direction, large-scale atmospheric circulation patterns (e.g., quasi-stationary Rossby waves^3^) form atmospheric linkages between different oceanic regions around the globe and control the spatial extent of the interactions among oceans^4^. These long-distance interactions, referred to as *teleconnections*, are marked by a significant correlation of the climatological variables. Teleconnections have periodicities ranging from months to several decades. Teleconnections have been identified to affect temperature, precipitation, storm tracks, severe weather, and droughts^5^. An in-depth knowledge of the dynamics and patterns of the different teleconnections is required for an advanced understanding in climate science which has direct impact on societies, economics and ecosystem.

Extensive research has analysed the teleconnections in the atmosphere and oceans considering a broad range of temporal and spatial scales, in both the tropics and the extratropics^6–8^. Among the most well-known teleconnections are a number of defined patterns that are marked by a strong correlation of the SSTs at different places like the Pacific Decadal Oscillation (PDO) and the North Atlantic Oscillation (NAO), but also the link between El Niño Southern Oscillation (ENSO) and the Asian monsoon system^7,9–12^.

Recent studies have raised concern that our understanding of climate processes remains incomplete, because interactions in multi-scale systems, like the climate system, are typically exhibited at unique or at multiple temporal and spatial scales^11,13,14^. Methods are required that help to unveil the interactions in the climate system at different scales, which usually remain hidden when looking at a particular scale only. Also, it is important to quantify the spatial distance of teleconnection variability between remote ocean regions at different timescales which could provide a quantitative understanding of short-range and long-range coupling between different oceanic regions.

To uncover such spatial and temporal variable interactions we use the complex network approach^15^, which recently has emerged as a powerful framework in extracting information from large high-dimensional datasets^16,17^. In addition, this non-parametric method allows investigating the topology of local and non-local statistical interrelationships^18^. Recently complex network analysis has facilitated the identification of spatial patterns in various research fields^19^. One such example are climate networks^18,20^ that are constructed from climate reanalysis data. Each grid cell of the climate dataset is considered as a network node and links between each pair of grid cells (or nodes) are setup using a similarity measures such as Pearson’s correlation coefficient^18,20^, event synchronization^21–24^, mutual information^14^, transfer entropy^25^ and multi-scale event synchronization^26^. Only the strongest links, filtered using either a predefined link density or fixed threshold, are retained in the final networks to help uncover the strongest interactions. Several network measures (such as degree, clustering, betweenness, community structures) have been used on the resultant climate network to capture local and global dependent climatic patterns within and among climate variables^23,27–29^, like global patterns of extreme rainfall teleconnections^14^ and spatial diversity of Indian rainfall teleconnections^30^.

To date, the complex network approach for the analysis of climate data has mostly been used to investigate patterns at one reference timescale only. However, the extension of the climate network to multiple timescales can reveal additional and important information as demonstrated recently^26,29,31^.

In this study, the wavelet-based correlation method is used by combining the wavelet transform and Pearson correlation^31^. We present a well-accepted climate network constructed from global monthly SST data, with a focus on identifying dependence structures on different timescales over the entire globe. In particular, we construct climate networks on eight different timescales, ranging from the monthly to interdecadal scale, using the wavelet-based correlation measure and interpret the detected short- and long-range links. Our findings re-confirm accepted knowledge about highly linked SST patterns like ENSO and PDO, but also provide new insights into the characteristics and origins of long-range teleconnections.

## Data and Methods

In this section, we describe the data characteristics used as well as methodological steps required for the construction of the networks.

### Data characteristics

The climate networks constructed are based on the global monthly sea surface temperature (SST) data provided by NOAA’s Earth System Research Laboratory (ESRL). The data (Extended reconstructed SST V3b) has a spatial resolution of 2.0-degree latitude x 2.0-degree longitude and is given for the time period 1979–2015. The data is freely available at NOAA’s portal (https://esrl.noaa.gov/psd/). As a preprocessing step we have removed 1056 grid points (out of total 10512) with missing values or gaps, hence in total 9456 grid points are considered. To avoid artefacts due to autocorrelation and seasonality, we removed the seasonal cycle and normalized the data. Specifically, we calculate for every month (i.e., separately for all Januaries, Februaries, etc.) the long-term mean and standard deviation. Each data point is then normalized by subtracting the mean and dividing by the standard deviation of the corresponding month at that grid cell. This normalization significantly reduces temporal autocorrelation in the time series^31^.

### Wavelet multiscale correlation

To construct the SST network, each SST grid cell is considered as a network node, and links between each pair of nodes are set-up based on a statistical relationship between them. The similarity measure used is the wavelet multiscale correlation (WMC) between the monthly anomaly series proposed by^31^. The proposed WMC method is a combination of two well established methods, the maximum overlap discrete wavelet transformation (MODWT) and the Pearson’s correlation. All the mathematical details are presented in the Additional information.

### Network construction

Formally, a network or graph is defined as an ordered pair G = {N, E}, containing a set of nodes N = {N_1_, N_2_, …, N_*N*_} together with a set E of edges {i, j} which are 2-element subsets of N. In this work, we consider undirected and unweighted graphs (G), where only one edge can exist between a pair of nodes and self-loops of the type {i,i} are not allowed. Hence, edges simply show connections between nodes, and each edge can be traversed in either direction. This type of graph can be represented by the symmetrical adjacency matrix^22,29^

1 $${A}_{i,j}=(\begin{array}{cc}1, & \{i,j\}\in E\\ 0, & \{i,j\}\notin E.\end{array}$$

**Figure 1:**
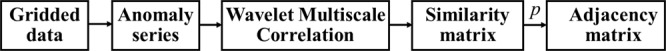
Schematic of network construction. Each grid cell of the SST dataset is considered as node and similarity between each pair of nodes is calculated using the WMC measure. By applying the 95*th* percentile threshold along with multiple testing, a link between each pair of nodes is set up.

To construct a climate network (Fig. 1), each grid cell of SST data is considered as a node of the network and undirected and unweighted edges are determined between all possible pairs of nodes based on their similarity. We use the WMC measure to quantify the similarity between the SST time series, yielding a square similarity matrix of 9456 × 9456 (total number of grid cells considered). We generate an adjacency matrix from the similarity matrix by applying a significance-based pruning which is explained in the next section.

### Multiple testing

A number of criteria have been proposed to generate an adjacency matrix from a similarity matrix, such as a fixed amount of link density^22,32^ or fixed thresholds^23^. Here, we consider a 5% link density since it is a well accepted criteria globally for the network construction. However, here we combine it with multiple testing attempts to avoid false links by controlling the type I error or adjusting *p*-values to give only significant links^33,34^. This is a very active area in statistics, and a range of methods have been proposed. Here, we use false discovery rates (FDR) to control *p*-values (for more details see Benjamini and Yosef  ^33^). This may seem like a stringent requirement (5% link density combined with multiple testing), but a large number of edges satisfies this criterion and are therefore retained in the network. To give a clear understanding of the final selected threshold, we tabulate all the threshold values (90*th*−, 95*th*−, 99*th* and FDR) in Table 1.

**Table 1:** Overview of sea surface temperature network threshold values (90*th*−, 95*th*−, 99*th* and FDR) at all timescales.

Percentile threshold	Original scale	1–2 months	2–4 months	4–8 months	8–16 months	16–32 months	32–64 months	64–128 months
90th	0.32	0.12	0.16	0.24	0.33	0.48	0.55	0.67
95th	0.41	0.17	0.22	0.32	0.44	0.61	0.66	0.76
99th	0.72	0.26	0.32	0.43	0.65	0.86	0.80	0.84
Multiple testing	Original scale	1–2 months	2–4 months	4–8 months	8–16 months	16–32 months	32–64 months	64–128 months
FDR	0.62	0.51	0.56	0.63	0.74	0.84	0.85	0.85

In this study we have selected threshold values obtained using False discovery rates (FDRs) method.

## Results

We present the results in three parts, first discussing the scale-specific spatial patterns obtained over different timescales, second investigating the occurrence of short- and long-range linkages between the identified scale-specific spatial patterns over different timescales, and finally providing a 3-D global visualization of the link distributions.

### Spatial patterns over different timescales

**Figure 2:**
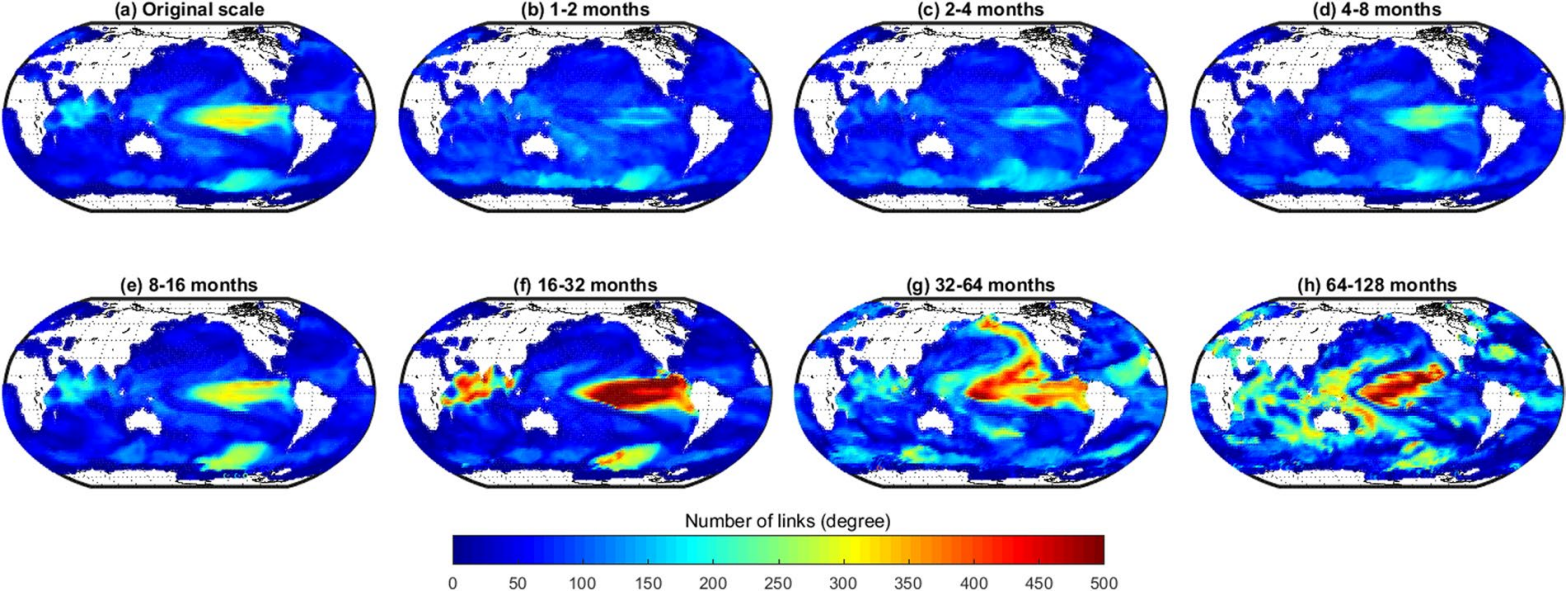
Number of links of each grid cell in a global SST network constructed at different timescales by considering only negative correlations.

Using the above-explained similarity measure and pruning method, we construct a separate SST network for each timescale (in total eight scales including the observed timescale, see Fig. 2). The networks reveal, separately for each timescale, how well the temporal evolution of the SSTs of each grid cell is linked to that of the other grid cells, i.e., it shows the number of significantly correlated edges ($\sum \,{A}_{ij}$, also called links) that each grid cell (or node) has. This gives us a basis for understanding what kind of teleconnections act at a certain timescale.

Considering only the positively correlated links (Fig. 2), it is obvious that at all scales the majority of the grid cells show a low link number that is larger than zero. We identify this as the background link number at a certain scale that is explained by the near-field spatial correlations of the grid cells^11,35^. In addition, there are distinct regions with a substantially higher number of links.

At the original, i.e., unprocessed scale (Fig. 2a) we observe a highly linked region in the mid to eastern equatorial Pacific accompanied by regions with a medium number of links in the southern Pacific, the western equatorial Pacific and the Indian Ocean. When looking at the scale-specific networks (Fig. 2b–h), it becomes clear that links at the observational scale are aggregated results of the coupling between scale-specific features of global SST data present at different timescales. Moving from lower to higher temporal scales, it becomes apparent that – with the annual cycle removed from the data – there are hardly any significant correlations between the SST evolution in different regions at timescales of less than a year (Fig. 2b–d).

However, at the 8–16 months scale (Fig. 2e), there are two zones with a relatively large number of significant connections in the mid to eastern equatorial Pacific and the southern Pacific. There is also a region with an above global average number of links in the Indian Ocean. While the number of links in this region increases when moving to the larger timescale of 16–32 months (Fig. 2f), the number of links from and to the Southern Ocean decreases.

At the scale of 32–64 months (Fig. 2g), the number of links in the mid to eastern Pacific Ocean decreases and a well-linked region in the form of a tilted horseshoe emerges in the northern Pacific, as well as a region with a medium number of links in the subtropical North Atlantic. On even longer timescales (64–128 months) the highly linked region in the equatorial Pacific re-emerges, as well as several smaller regions with a medium number of links in all three ocean basins (Fig. 2h).

**Figure 3:**
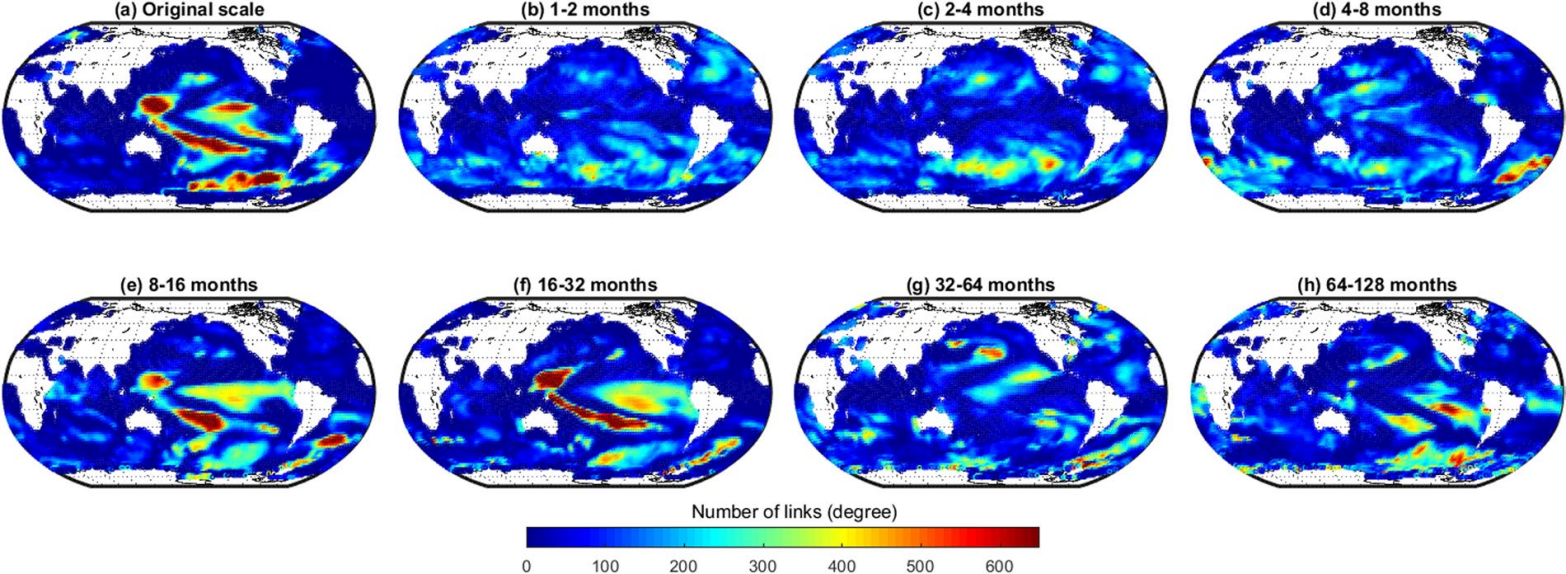
Number of links of each grid cell in a global SST network constructed at different timescales by considering only positive correlations.

Figure 3 shows the number of negatively correlated links. In contrast to the positive ones (Fig. 2), there is no global background link number due to the fact that the near-field correlation is in general positive. On the original scale, regions whose SST evolutions are negatively correlated to that of other regions can mainly be found in the Pacific Ocean (Fig. 3) centered around the equator as well as in the Southern Ocean. The separation in the different timescales again reveals that the observed patterns are aggregates of the timescale-specific feature. Again there are only a few highly linked regions on timescales of less than a year (Fig. 3b–d) with the exception of the subtropical South Atlantic (Fig. 3d). On longer timescales of about one to two years the link number in the South Atlantic stays rather stable and highly linked regions in the Pacific emerge (Fig. 3e,f), namely in the mid to eastern equatorial Pacific and the equatorial to south-western Pacific. The latter becomes more linked on the 16–32 months timescale, while the link numbers of the former decrease. Moving to interannual timescales, the regions with a large number of significantly negatively correlated edges globally decrease but a few regions in the South Pacific remain (Fig. 3h).

### Occurrence of short- vs. long-range links

**Figure 4:**
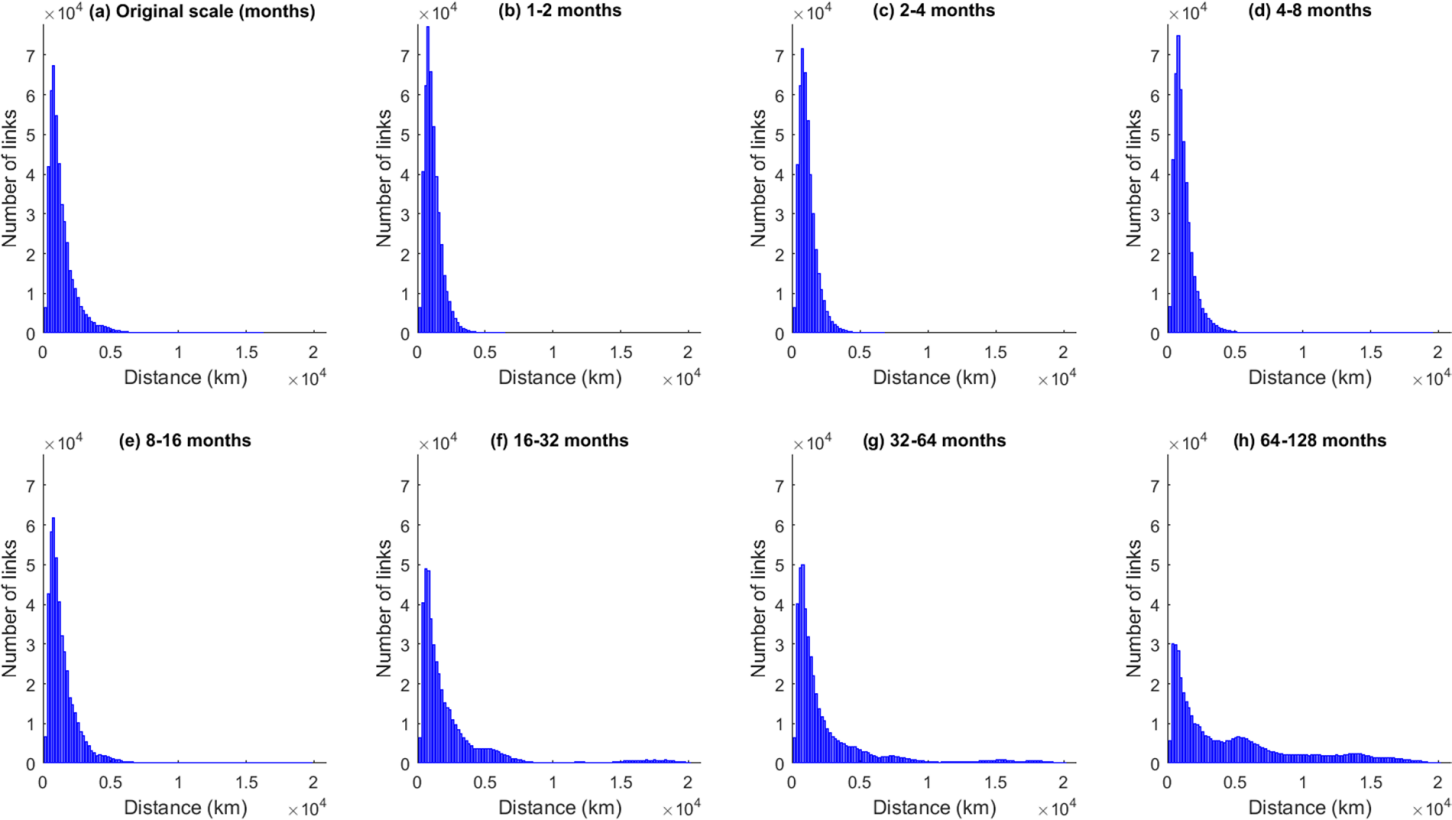
Frequency distribution of the link lengths in SST networks at different timescales. Short-distance links can be interpreted as near-field correlations, while long-distance links suggest long-range spatial dependencies or teleconnections.

We further investigate the length distribution of the significantly correlated edges (both positive and negative) at the different scales by creating histograms showing the number of links plotted over the geographical length link (Fig. 4). The geographical link length (shortest geographical distance between two points on a globe) was computed using Haversine’s formula which determines the great circle distance between two spatially embedded points on the sphere^36^.

For all scales (Fig. 4a–h) these frequency distributions of the link lengths show that the dominant factor controlling the significance of a correlation between two SST time series is the near-field spatial correlation. This property is embodied by Tobler’s First Law of Geography, which states that ‘everything is related to everything else, but near things are more related than distant things’^37^. Nevertheless, the number of links decreases again at very short distances. There are two reasons for that: First, because the nodes are evenly spaced angularly on the sphere, neighboring grid points near the equator are much further apart (>500 km) than those near the poles. Second, the maximum number of possible links depends on the geographical link length due to the spherical shape of the Earth and the land ocean distribution on it; in general, the number of possible links increases with the link length until a maximum at a length of about 10,000 km and then decreases again. The second noticeable feature is a ‘fat tail’ for the longer timescales (16–32 months and longer, Fig. 4f–h) that suggests the presence of teleconnections, i.e., a similar time-dependent behavior in locations that are geographically separated.

**Figure 5:**
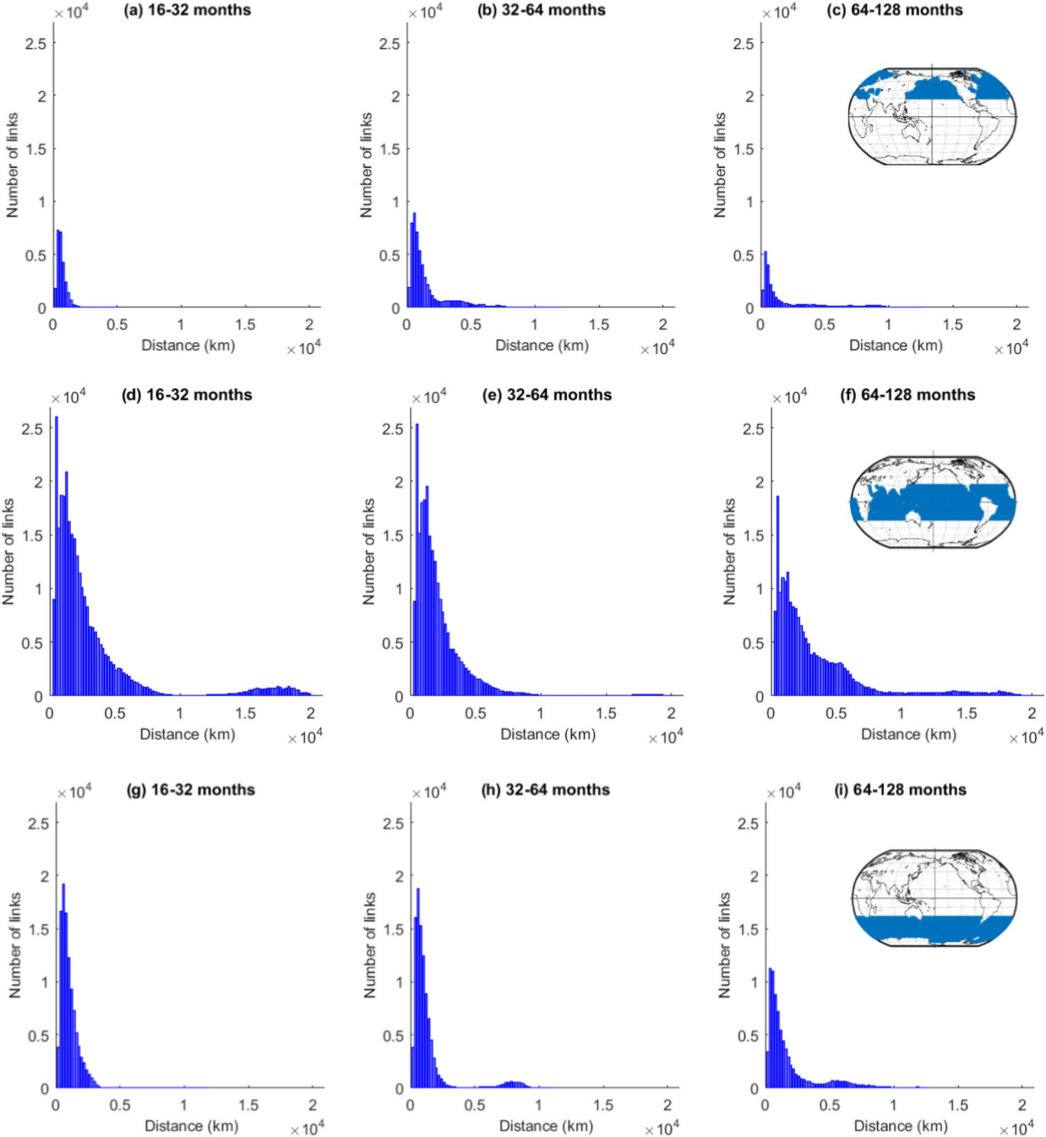
Frequency distribution of the edge lengths in SST networks at different timescales within three standard zones, namely NH, TH and SH (within-region links). Short-distance links show near-field spatial correlation, while long-distance links suggest long-range spatial dependencies or teleconnections within the same zone.

To better understand the nature of these teleconnections, we divide the entire globe into three standard regions, namely the extratropical Northern Hemisphere (NH), the Tropical Hemisphere (TH) and the extratropical Southern Hemisphere (SH), and analyse separately the frequency distributions of the link lengths of edges that connect nodes only within these regions for timescales larger than 16–32 months (Fig. 5).

It can be seen that short-range links appear in all regions on all the considered timescales (i.e. larger than 16 months) with the smallest number of short-range links occurring at the very long timescales (64–128 months) for all three hemispheres. When looking at the long-range links, the three hemispheres show a different behaviour.

In the tropics, teleconnections appear on timescales from 16–32 months and on the longer timescales of more than 64 months. At these timescales, there are also long-range links in the two extratropical hemispheres that start developing at the 32–64 months timescale at which the tropics show no long-range links and additionally a notable reduction in short-range links. The geographical lengths of the teleconnections in the tropics can be longer than those in the extratropics due to Earth’s spherical shape as grid points in the tropics have larger distances. Hence, we conclude that the length distribution of links varies between the different decadal timescales for the global and the within-region analysis.

**Figure 6:**
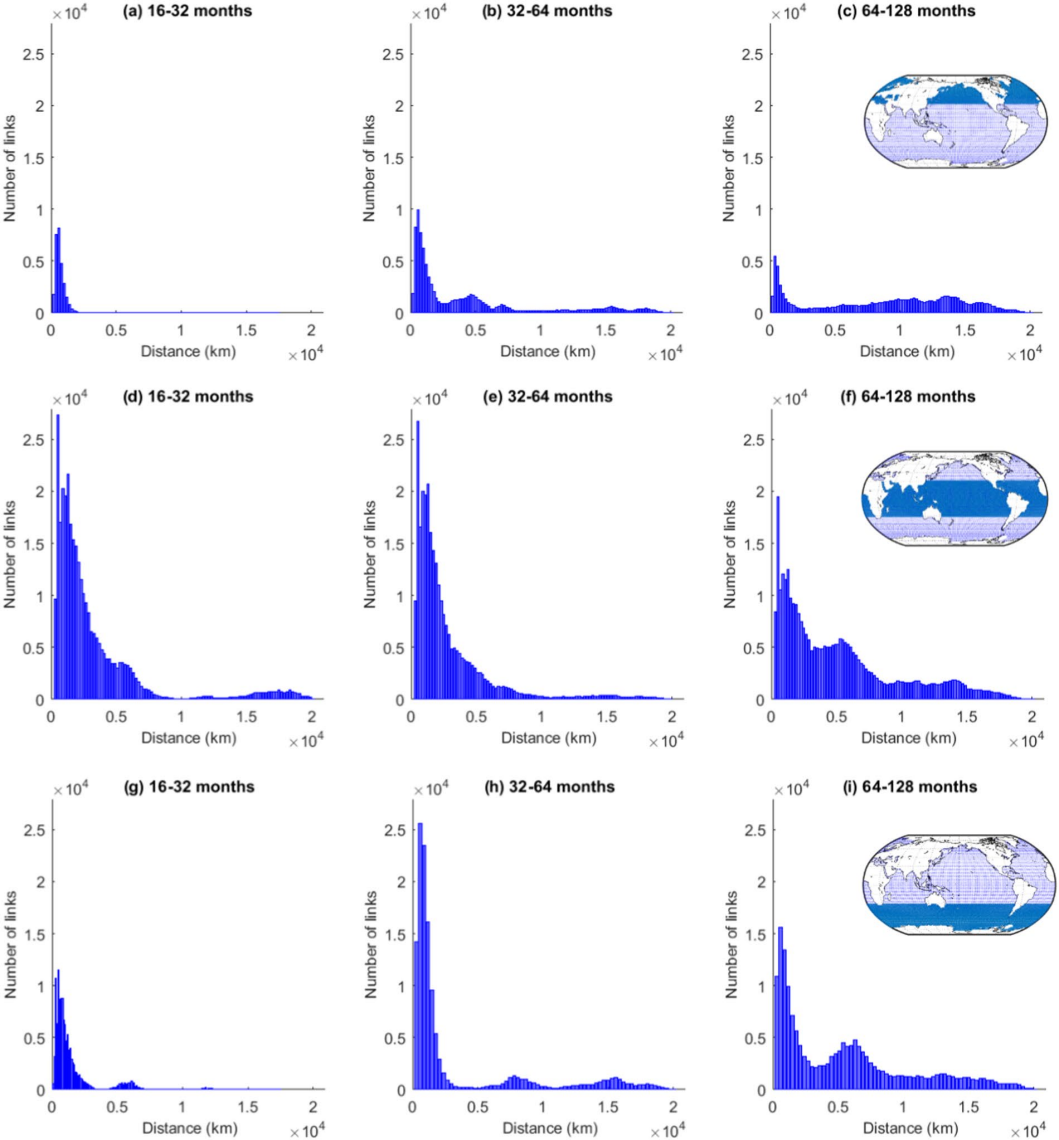
Frequency distribution of the edge lengths in SST networks at different timescales constructed for standard zones, namely NH, TH, and SH, considering the interaction with the whole globe (beyond-region links). Short-distance links show near-field correlation, while long-distance links suggest long-range spatial dependencies or teleconnections.

As most of the teleconnections that we are interested in act across the three zones, we further analyse the link length frequency distribution from one region (NH, TH, and SH) to the rest of the earth (including within-region links, Fig. 6). This will help us to understand the interactions between tropics and extratropics which cannot be revealed by Fig. 5.

Figure 6 confirms that the extratropics (NH & SH) do not possess teleconnections at the interannual scale (16–32 months) both within the same region (shown in Fig. 5) and with the rest of the earth (Fig. 6). We also notice that the absolute number of short-range connections (Fig. 6) at this scale remains almost unchanged in all three regions except for slight increments in the extratropical SH which depict the near-field interactions between SH and TH. These links connect regions with a distance of about 5,000 km, which is visible in Figs 2 and 3.

The first interesting observation at the intradecadal scale (32–64 months) is the significant increase in short-range connections in the extratropics (NH and SH) in contrast to almost no significant changes in the tropics compared to the interannual scale (Fig. 6). However, Fig. 5e already confirmed that the tropics do not have much within-region short-range connections at the 32–64 months scale compared to the 16–32 month scale (Fig. 5d). Also the increase in short-range connections in NH is mainly credited to internal short-range connections (Fig. 5b). These observations suggest that SH exhibits a significant number of short-range links with the tropics at the intradecadal scale. Moreover, we find that long-range connections appear in the extratropics (NH & SH) which are completely absent at the interannual and other finer scales (Fig. 6a,c) or when looking only at the within-region links (Fig. 5b,h). This results indicates that interactions in the climate system occur at specific timescales but are absent at other timescales, which undermines the necessity of multi-scale analyses.

At the decadal scale (64–128 months) and for all three zones, we observe a significant reduction in beyond-region, short-range connections compared to the intradecadal scale. For NH, there is the same reduction as is seen for within-region links, whereas the reduction is larger for SH and TH. This observation indicates a decline in near-field connections within NH and in-between TH and SH. The rise in NH and SH at distances of about 10,000 to 15,000 km shows the significant coupling of the two hemispheres to the other regions. The fact that for both extratropical hemispheres the number of very long links increases, while it stays rather constant for TH, indicates the existence of very long-range links between SH and NH.

These analyses uncover that (i) at the interannual scale the tropics show the largest number of significant correlations acting over both short and long distances and the existence of links with a length of about 5,000 km between SH and TH (Fig. 6d,g); (ii) at the intradecadal scale the main characteristics are long-range links between NH and TH with a length of about 5,000 km and 7,000 km (Fig. 6b,e), as well a small number of links between SH and TH with a length of about 15,000 km, yet there are no long-range links within TH; (iii) at the decadal scale long-range connections between NH and SH to the tropics as well as interhemispheric (SH to NH) links exist.

### 3-D visualization of the link distributions

The results listed above identify the ocean regions that are most closely linked when looking at the variability of SST at different timescales going from the intra- to interannual and even decadal scale (Figs 2 and 3). The analysis of the frequency distributions of the link lengths gives further insight into the occurrence of teleconnections (Figs 4–6). What is missing is a detailed knowledge about the actual regions that are linked at the different timescales.

**Figure 7:**
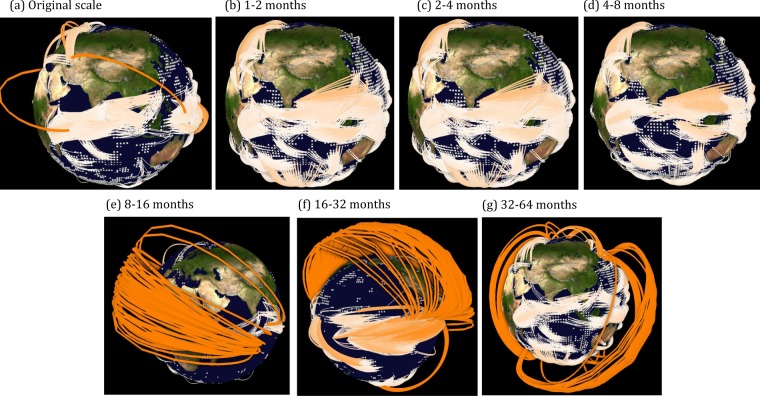
Spherical 3-D globe representation of the short- and long-range connections in SST networks at different timescales. Only nodes beyond a pre-selected betweenness centrality (BC) value are plotted. For instance, in subplot (**a**–**d**),(**e**–**f**) and (**g**) nodes are plotted for BC values greater than 90 *K*, 57 *K* and 38 *K*, respectively. Edge color represents the geographical link length.

We, therefore, visualize the climate networks on spherical 3-D node-link maps (Fig. 7) constructed at different timescales, where the node positions are fixed, and links are plotted using the software GTX tool. To best visualize the structures of the global climate network, especially the connectivity of different regions either via short- or long-range links, we do not plot all links but focus on the regions that were identified as being highly linked. The 3-D projection of the networks reveals patterns consistent with the 2-D visualization (Figs 2 and 3). However, it contains additional information about short- and long-range connections, e.g., even though the 2-D visualization at the original scale (Figs 2a and 3a) depicts approximately the same patterns as the 16–32 months scale (Figs 2e and 3e), the 3-D visualizing clarifies that there are hardly any long-range connections present at the original scale (Fig. 7a) compared to the 16–32 month scale (Fig. 7e). This visualization of the connections helps in attributing the interrelationships between selected regions at certain timescales. Thus, the node-link maps add valuable information to the discussion and interpretation of the results obtained in previous Sections.

## Discussion

In the following we link our results to known modes of climate variability and teleconnections and highlight the new findings of this study.

The link maps (Figs 2 and 3) clearly reveal the known modes of SST variability on the various time-scales. The highly linked region in the eastern equatorial Pacific can be identified as El Niño/Southern Oscillation (ENSO), a periodic fluctuation in SST (accompanied by a fluctuation in air pressure) across the equatorial Pacific, that occurs with an average time between peak events of about four years^38^ and can therefore be seen best at the 16–32 month scale (Fig. 3f). At the same timescale, the region of variability of the Indian Ocean Dipole (IOD) shows a large link number, as IOD events acts on about the same timescale as ENSO^39^. ENSO and IOD are known to impact each other via the atmosphere^40–42^, and the node-link map reveals that the links between the two modes exist mainly between the northern part of the ENSO tongue and the equatorial Indian Ocean (Fig. 7e,f). Furthermore, the analysis exhibits that the arrow shaped pattern of negatively correlated links in the western equatorial Pacific (Fig. 3f) that occurs at the same timescale as El Niño is neither linked to ENSO nor to IOD.

The last prominent feature at the intraannual timescale is the highly linked area in the Southern Ocean (e.g., Figs 2f and 3f). Our 2-D visualization confirms here what has just been analysed by Ferster *et al*.^43^: a link between SST in the Southern Ocean to ENSO events via the Southern Annular Mode (SAM), i.e. the north–south movement of the westerly wind belt that circles Antarctica. Positive states of SAM correlate with a cooling of SSTs in the high-latitude Southern Ocean, and a warming within the Southern Hemisphere sub-tropics and mid-latitudes^43^; this negative correlation can be seen in in our 2-D visualization (Fig. 3f). Our 3-D visualization further uncover a teleconnection between the very eastern part of ENSO and the region in the Southern Ocean near Western Antarctica (Fig. 7). From the 2-D maps it follows that this teleconnection has to be positively correlated (Fig. 2f).

Going to longer timescales (32–64 months) we find the majority of the highly linked regions in the northern Pacific. The form and location of these regions resemble strongly the pattern of the Pacific Decadal Oscillation (PDO), a pattern in SST and sea-level pressure that varies between positive and negative phases on periods of 20–30 years. Yet we find high link numbers at the interannual scale. A detailed look shows that this is not a contradiction to the known facts: Viewing the 3-D visualization (Fig. 7g) we find that most of the links are not within the PDO pattern (these indeed should act on a 20–30 year scale), but are teleconnections to the Southern Ocean. These can act on shorter timescales, likely via a western Pacific ocean-atmosphere pathway, whereby SST anomalies can propagate from the southern to the northern hemisphere^5^.

But not all teleconnections work via the atmosphere. The 3-D visualization (Fig. 7g) shows a number of links going from the North Atlantic to the South Atlantic (this can also be seen in Fig. 3f). This negative correlation likely shows the so-called see-saw response^44^ that is due to the transport of heat from the Southern Ocean to the North Atlantic via the Atlantic Meridional Overturning Circulation (AMOC). If the AMOC is stronger (as it has been in the period after year 2000 compared to the years before^45^), more heat is transported towards the north which leads to a cooling in the Southern Ocean and a warming in the subpolar North Atlantic. (The link diagram for the next ‘64–128’ months scale is not available, since the network size has grown to an extent which was unable to process in the used software.)

More general information on where and at what distances the teleconnections act is given by the histograms of the frequency distributions of the link lengths. Within one region we find teleconnections mainly in the tropics (Fig. 5d) acting on a length scale of about 18,000 km. There are also a few long-range links in both the extratropics NH and SH, but here on all timescales the short-range links dominate. This is different when looking at the links from one region to the others (Fig. 6). Here a number of long-range links are detected, especially on the 64–128 month scale on spatial scales of about 6,000–7,000 km as well as about 10,000–15,000 km. This is likely due to the fact that teleconnections often work via Rossby waves that link the tropics to the extratropics. The wavelengths of these Rossby waves depend on the strength of the westerly winds, but are typical about 7,000 km for weak westerly flow in the mid-latitudes and increase for stronger winds and more polar regions^46^.

In general, we can conclude that our method enables us to distinguish between regions that are highly linked through significantly correlated short-range edges and those that have long-range teleconnections. While the former are typical for proximity-based correlation on very short timescales as well as for correlations within the region where a climate mode takes place (like El Niño or the Indian Ocean dipole) and are mainly due to the advection of heat by the oceanic surface, the long-range links correspond to teleconnection patterns that either act via the atmosphere, e.g., through Rossby waves, or through the major open circulation systems like the AMOC. The latter warrant even more investigation as they are of particular importance for the understanding and forecasting of lasting weather events like droughts, flooding and temperature extremes.

## Conclusions

Ocean-atmospheric teleconnections are intriguing phenomena in the climate system as they connect very remote regions. The thorough diagnosis of their characteristics will improve our understanding of the climate system with large potential benefits, such as improvement in seasonal forecasting of climate variables and weather extremes. Here, we propose a unified framework to investigate and unravel short-range and long-range teleconnections at different timescales and spatial distances based on wavelet multi-scale correlation and give a first insight into the potential usage of this method. The study detects the highly linked regions in the oceans at different scales along with the identification of the link type, i.e. spatial proximity connections, short-or long-range teleconnection. In addition, the proposed method allows identifying the regions that are highly linked within themselves regarding their SST evolution and distinguish them from regions that have long-range teleconnections to other ocean regions. It unravels, for example, which parts of the well-known ENSO SST pattern in the equatorial Pacific are intralinked and from which regions the teleconnections with other climatic modes originate. This adds valuable information to the understanding of the functioning of teleconnections. The method has further potential to understand how far a particular teleconnection has an atmospheric or oceanic origin. This could be worthwhile for regional climate modeling since it highlights the regions that are necessary to include when modeling climate variability at a certain scale.

### Wavelet multi-scale correlation

MODWT decomposes the time series into different time scales or frequency components. The wavelet decomposition is realized using the two basis functions known as father wavelet ($\varphi (t)$) and mother wavelet ($\psi (t)$). The general admissibility conditions for $\psi$ to be called a wavelet function are

2 $${\int }_{-\infty }^{\infty }\,\psi (t){\rm{d}}t=0$$

3 $${\int }_{-\infty }^{\infty }\,\psi {(t)}^{2}{\rm{d}}t=1$$

Any function *f*(*t*) can be expressed through these basis functions and their scaled and translated versions are

4 $$\begin{array}{rcl}f(t) & = & \sum _{k}\,{s}_{J,k}{\varphi }_{J,k}(t)+\sum _{k}\,{d}_{J,k}{{\rm{\Phi }}}_{J,k}(t)\\  &  & +\,\sum _{k}\,{d}_{J-1,k}{{\rm{\Phi }}}_{J-1,k}(t)+\cdots +\sum _{k}\,{d}_{1,k}{{\rm{\Phi }}}_{1,k}(t)\end{array}$$

where *J* is the total number of scales to be analyzed, and *k* is in the range of 1 to *l* (length of the time series). The coefficients *S*_*J*,*k*_, are the approximation coefficients and *d*_*J*,*k*_, …, *d*_1,*k*_ are the wavelet transform coefficients at scales *J* to 1, while the functions ${\varphi }_{J,k}(t)$ and $\{{{\rm{\Psi }}}_{j,k}|j=1,\ldots ,J-1,J\}$ are the basis functions which are obtained through translation and dilation of the father of father ($\varphi (t)$) and mother ($\psi (t)$) wavelets function respectively.

The mother and father wavelet is scaled (or dilated) by a factor *j* and translated (or shifted) by a factor *k* to give

5 $${\varphi }_{j,k}(t)={2}^{\frac{-j}{2}}\varphi ({2}^{-j}t-k)$$

6 $${{\rm{\Phi }}}_{j,k}(t)={2}^{\frac{-j}{2}}{\rm{\Phi }}({2}^{-j}t-k)$$

Further, the values of the wavelet transform coefficients at each of the scale and the approximation coefficients at scale *J* are estimated by:

7 $${d}_{j,k}\approx \int \,{{\rm{\Phi }}}_{j,k}(t)f(t)dt,\,j=1,\ldots ,J-1,J$$

and

8 $${s}_{j,k}\approx \int \,{\varphi }_{J,k}(t)f(t)dt$$

where the scaling coefficients *s*_*J*,*k*_ capture the smooth trend of the time series at the coarse scale 2^*J*^, which are also called smooth coefficients; and the wavelet coefficients *d*_*j*,*k*_, also known as detail coefficients can detect deviations from the coarsest scale to the finest scale.

The original series *f*(*t*) can be reconstructed by summing up the detailed components and the smooth components:

9 $$f(t)={S}_{J,k}+{D}_{J,k}+{D}_{J-1,k}+\cdots +{D}_{1,k}$$

where $${S}_{J,k}={\sum }_{k}\,{s}_{J,k}{\varphi }_{J,k}(t)$$; $${D}_{J,k}={\sum }_{k}\,{d}_{J,k}{{\rm{\Phi }}}_{J,k}(t)$$; $${D}_{1,k}={\sum }_{k}\,{d}_{1,k}{{\rm{\Phi }}}_{1,k}(t)$$.

Further, consider two time series {*X*(*t*)} and {*Y*(*t*)} with the same length *T*. The wavelet multi-scale correlation (WMC) measure between both time series can be estimated as^47,48^

10 $$WMC={\rho }_{X,Y}^{{l}_{j}}\equiv \frac{CO{V}_{X,Y}^{{l}_{j}}}{Va{r}_{Y}^{{l}_{j}}Va{r}_{X}^{{l}_{j}}}$$

where

11 $$Va{r}_{X}^{{l}_{j}}=\frac{1}{{T}_{j}}\,\sum _{t={M}_{j}-1}^{T-1}\,{[{d}_{j,t}^{X}]}^{2}$$

12 $$Va{r}_{Y}^{{l}_{j}}=\frac{1}{{T}_{j}}\,\sum _{t={M}_{j}-1}^{T-1}\,{[{d}_{j,t}^{Y}]}^{2}$$

13 $$CO{V}_{X,Y}^{{l}_{j}}=\frac{1}{{T}_{j}}\,\sum _{t={M}_{j}-1}^{T-1}\,{d}_{j,t}^{X}{d}_{j,t}^{Y}$$

${d}_{j,t}^{(.)}$ denotes the MODWT wavelet coefficient of variables {*X*, *Y*} at scale *l*_*j*_; ${T^{\prime} }_{j}=T-{M}_{j}+1$ stands for the number of coefficients unaffected by the boundary; ${M}_{j}=({2}^{j}-1)\,(M-1)+1$ represents the length of the scale *l*_*j*_ wavelet filter, and *M* is the width of the wavelet filter. The multi-scale correlation measure ${\rho }_{X,Y}^{{l}_{j}}$ denotes the scale-wise correlation between *X* and *Y* at different *l*_*j*_ scales. Like the Pearson correlation coefficient (PCC), the value of ${\rho }_{X,Y}^{{l}_{j}}$ ranges between −1 and 1. ${\rho }_{X,Y}^{{l}_{j}}=0$ implies the variables *X* and *Y* are not correlated at scale *l*_*j*_. ${\rho }_{X,Y}^{{l}_{j}}=1$ and ${\rho }_{X,Y}^{{l}_{j}}=-\,1$ indicate that the variables are perfectly correlated and anti-correlated, respectively. The values of ${\rho }_{X,Y}^{1{l}_{j}}$.….${\rho }_{X,Y}^{{l}_{j}}$ indicate the strength of the relation between *X* and *Y* at different temporal scale bands.
